# Supplementary material for: An assessment of the global impact of 21st century land use change on soil erosion
Source: Nat Commun. 2017 Dec 8;8:2013. doi: 10.1038/s41467-017-02142-7 (PMC5722879; doi:10.1038/s41467-017-02142-7)
Supplement: Supplementary file 1 — Supplementary Information [file 41467_2017_2142_MOESM1_ESM.pdf]

Supplementary Note 1 | Estimating soil loss by USLE/RUSLE

Erosion-prediction technology is established when a set of mathematical equations is used to compute the values of soil erosion variable using input values for climate, soil, topography and land use.

Many mathematical models, categorized as empirical, conceptual, physically-based or process-oriented are available to estimate soil erosion at different spatial and temporal scales[1,2]. At present, the research community is contemporaneously working on both improving the applicability of complex process-oriented models[3–5] and updating the existing empirical approaches such as the Universal Soil Loss Equation (USLE)[6–12] which remains attractive from a practical point of view[13,14].

Choosing of the soil erosion model to be applied, it has to be taken into account that it is not always necessary to calculate the exact erosion rate for a particular situation[15] but rather compare among different situations. Choosing which model to apply becomes a matter of data availability and what type of information has to be obtained. As an example, Nearing[15] noted that most applications of WEPP were developed in the US because of the availability of soil, climate and crop information for that area. Another example was given by Hann and Morgan[16] who adapted USLE to indicatively predict ground cover effects on soil loss due to an erosive event with a given return period, although USLE is not recommended for the prediction of specific soil loss events[17].

The Universal Soil Loss Equation (USLE) is a good example of grey-type model[3,18], in which the relationship between soil loss, rainfall erosivity and soil type is corrected using information on slope steepness, slope length, crop cover and anti-erosive measures management. Combining process-based models, such as WEPP with empirical ones allowed to relate, as an example, sheet and rill erodibility to nomograph estimates of the soil erodibility factor of the USLE[19].

The USLE was developed at the National Runoff and Soil Loss Data Center, in cooperation with Agricultural Research Service and Purdue University[6]. It resulted from statistical analysis of more than 10,000 plot-years of basic runoff and soil loss data[20] carried out in plots having a length less than or equal to 122 m and a slope ranging from 3 to 18%. Defining the mathematical structure of the USLE a reference condition, named as *unit plot*, was also used. The unit plot was defined as a plot 22.1 m long, with a 9% slope, maintained in a continuous regularly tilled fallow condition

with up-and-down hill tillage. The unit plot was used to compare soil loss data collected on plots that had different slopes, lengths, cropping and management and conservation practices.

USLE/RUSLE was originally designed to predict long-term average annual soil loss[6] associated with sheet and rill erosion. For this reason the model tends to over-predict small annual soil losses and under-predict large annual soil losses[21] although more process-oriented models like WEPP have the same performance[22,23].

At present, USLE and RUSLE are by far the most applied soil erosion prediction models. According to Risse *et al.*[21] '*USLE has been used throughout the world for a variety of purposes and under many different conditions simply because it seems to meet the need better than any other tool available'*. By an ISI query (http://isiknowledge.com/) for the period 2003-2014, Auerswald *et al*.[24] stated that 844 hits correspond to the keywords 'Universal Soil Loss Equation', 'USLE', 'Revised Universal Soil Loss Equation' and 'RUSLE'. Our own ISI query for 2003-2016 resulted in 1,118 hits, which indicates a steep increase over the last years (2013: 97, 2016: 129 with 8 papers highly cited and 3 'hot papers'). The most well-known models based on USLE/RUSLE technology, such as SWAT[25], AGNPS[26], Watem/Sedem[27], EPIC[28] lead to 243 hits in the same period (2003-2014). Approaches independent of the USLE technology, such as WEPP[29], LISEM[30], EUROSEM[31] and PESERA[32] result in 254 hits.

Stolpe[33] applied RUSLE, EPIC and WEPP at the Mediterranean climate of Chile. His results show that RUSLE and WEPP models provide the same accuracy in soil loss estimation.

Furthermore USLE still represents the best compromise between applicability in terms of required input data and reliability of obtainable soil loss estimates[21]. It has become the standard technique of soil conservation workers[1].

USLE predicts long-term average annual erosion by water at an acceptable level of reliability[34] also in areas of Western Europe[8,9,35].

Notwithstanding its empirical origin, Ferro[36] demonstrated that the original structure of USLE can be theoretically obtained applying the dimensional analysis and the self-similarity theory[37,38] using the same soil erosion representative variables and the reference condition adopted by Wischmeier and Smith. In other words, applying the factor scheme and the reference condition adopted by Wischmeier and Smith, Ferro[36] overcame the empirical origin and limits of the model since he

stated that '*USLE is the subsequent logical structure with respect to the variables used to simulate the physical soil erosion process*'.

USLE, RUSLE and WEPP (or other process-oriented models) constitute a complementary suite of models useful to achieve the specific user need[15].

The choice of a soil erosion-prediction technology is also dependent on the spatial and temporal scale of model application. Process-oriented models require an application of the used equations at a given spatial scale, ranging from plot to basin, and at event temporal scale. Event and at-point or small scale (hillslope) models are not suitable to be applied for simulating soil loss on a wide region.

At a large spatial scale, the area has to be discretized using, for example, a square grid subdivision (raster scheme), choosing a mesh size consistent with the scale of the original model deduction. Using a raster scheme applied to the USLE model corresponds to hypothesize that each cell is independent of the others with respect to soil loss. In other words, at large spatial scale such as a region or a global perspective, a simple index-based model able to calculate an average soil loss, at annual or mean annual scale, allows to compute the involved factors using spatially distributed input values. The cells cannot be assumed to be independent from each other when sediment delivery processes have to be modeled such as at basin scale. Even in this case, the USLE scheme can be applied by coupling it with a mathematical operator expressing the hillslope transport efficiency[39,40].

Obtaining accurate and reliable soil loss estimate using spatially distributed models on wide regions depends on both the resolution (vertical and horizontal) of the input topographic information and the quality of the land-use input data.

USLE/RUSLE is a good compromise for wide region application when both a mesh-size comparable with the original developed slope-length scale is applied and information on rainfall, soil and land-use systems is available.

Improving interpretation of the soil erosion processes at different spatial and temporal scales and testing or developing physically oriented modeling approaches have an obvious scientific importance. Simple and physically plausible empirical methods for predicting soil erosion are the ones allowing soil erosion prediction by professionals and technicians. Soil loss estimate are

considered reasonably accurate for most practical purposes, especially for wide spatial scale applications, when the errors of the predictions do not exceed a factor of two or three[35]. Improvement of empirical models is committed to simply describe runoff processes since erosion is a runoff-driven process[11,23]. There are many signs that this USLE-based approach is very useful for estimating high soil losses[41]. This is of particular interest for both an effective soil conservation design and an accurate prediction of pluri-annual soil loss values since a few large events affect total soil loss for an area of interest[34].

Supplementary Note 2 | Definition of soil erosion

For the avoidance of doubt, in this study the term soil erosion refers to RUSLE soil loss (i.e., an estimate of the soil loss due to sheet and rill erosion processes). The RUSLE ‘*Soil loss refers to the amount of sediment that reaches the end of a specified area on a hillslope that is experiencing net loss of soil by water erosion. It is expressed as a mass of soil lost per unit area and time. There are several aspects of erosion that are implied in this definition. First of all soil loss refers to net loss, and it does not in any way include areas of the slope that experience net deposition over the long term. As such, soil loss does not equate to the sediment yield from a hillslope that exhibits toe-slope deposition, which are most cases. It is, rather, the sediment delivered to the bottom of the slope area that feeds onto the toe slope*.’[42]

Supplementary Note 3 | Sensitivity analysis

Sensitivity analyses (SA) evaluate the influence of parameter estimation on uncertainty and the effect of parameters on model output[43]. Two main branches of SA are Local Sensitivity Analysis (LSA) in which the effect of every single factor on model prediction is analyzed, and Global Sensitivity Analysis (GSA) where higher order interactions among factors are considered when evaluating factor importance and their combined effect on model output[44].

In this study, a modified version of the approach by Harper *et al*.[45] was applied. Random Forests (RF) were used in order to estimate variable importance, however RF mapping-function is most often high dimensional and therefore difficult to visualize and interpret, so simple variable importance ranking is usually computed. A more descriptive summarization of the RF can be obtained using Feature Contribution (FC). FC decomposes a RF mapping structure into additive components. Plotting FCs against variables values yields plots similar to marginal-effect plots. This allows to separate the main effects and identify and quantify interactions. Supplementary Fig. 5 depicts the effect of each factor on the estimated soil erosion (expressed on logarithmic scale, vertical axis) versus each one of the influencing factors; the variables are ranked according to their importance, expressed as R^2. The color scale expresses the value variation of the most influent variable (cover-management factor (C)) and allows to evaluate the interactions among variables (for instance high values of C correlate with lower values of the rainfall-runoff erosivity factor (R) and high values of soil erodibility factor (K). The plot was obtained using out-of-bag cross validated replicas of the larger dataset, each point in the plot is the averaged out-of-bag value of these replicas.

The second part of the GSA approach by Harper *et al*.[45] consists of using Classification and Regression Trees (CART) to analyze and visualize the interactions among variables. In this study, the authors constrained the tree to a depth of three nodes (Supplementary Fig. 6), in order to maintain the output to a lower level of complexity and to make the output understandable. The tree plot shows the influence of the most important parameters (C, LS, R) and how their interaction affects the estimated soil erosion (shown as a boxplot at the terminal nodes level). One of the advantages of using a partition model as CART is that the node membership can be spatially mapped. Supplementary Fig. 7 shows the node membership as a map, where each pixel in the map is attributed to the most frequent node class in pixel. It is rather evident that many nodes are influenced by vegetation cover (implied by the importance of the C factor), with node five

representing mostly tropical forests and node four boreal forests. Other nodes are influenced by rainfall, with nodes seven and eight differentiating between forest cover with low and high rainfall intensity. Nodes eleven and twelve are influenced by topography and climate, with eleven corresponding to areas with little rainfall intensity and flat topography, while twelve have similar small rainfall intensity, but are characterized by mountainous or hilly topography. Finally, nodes fourteen and fifteen express areas of average rainfall intensity, but differentiate between more different soil cover.

Previous studies dealing with the importance of each RUSLE factor in model uncertainty at plot level, observed that the C-factor is the most sensitive model parameter in predicting soil erosion[21,46,47]; followed by the LS-factor. This order of importance of the RUSLE factors is confirmed by plot as well regional level applications[48]. However, sensitivity assessments on model run at catchment level highlighted different scenarios, with RUSLE predictions being most sensitive to the rainfall-runoff erosivity factor (R-factor)[49] and the topographic factor (LS-factor)[50,51]. The order of the most important factor driving RUSLE soil erosion predictions seems to depend on specific environmental conditions. Sensitivity analysis[49] in a subtropical zone characterized by very high R-factor[52] supports this hypothesis. On the basis of this sensitivity analysis, it was observed that the soil erosion predictions of the global RUSLE-based model are most sensitive to the C-factor and to a lesser extent to the R-factor and the LS-factor. According to previous studies at different scale (plot, catchment and regional), the sensitivity analysis confirmed that soil erodibility is by far the least sensitive model component. The order of factor interactions obtained from the CART analysis allowed to highlight the local sensitivity conditions associated to the global RUSLE-based soil erosion assessment presented in this study Supplementary Fig. 7.

Supplementary Note 4| Uncertainty analysis

The RUSLE is a purely deterministic model in which the product of physical measures is used to derive the amount of soil loss. As such, a rigorous assessment of uncertainties is not feasible, nor would it be meaningful, unless the uncertainties of the input layers and their propagation in the model scheme were quantified. Accordingly, the estimation of the uncertainty in the RUSLE model outputs remains in most case an unaddressed issue[51]. A thorough quantification of uncertainty associated to the RUSLE model was provided only in a few local-scale studies, mainly dealing with a single model factor such as rainfall[53], soil type[54] and topography[55].

However, in a global scale application most (if not all) of the spatial layers used to derive RUSLE variables lack information about the associated uncertainty. For instance the SRTM DEM, used to calculate the LS factor, misses the adequate spatial information about the land-cover-, latitude- and elevation-depending uncertainty of the data. For this reason, it is impossible to use uncertainty propagation techniques to estimate uncertainty.

Previous RUSLE-based global studies[56], proposed a different approach[57] based on the introduction of random errors in the input layers, with the propagation of this random noise in the final value of soil loss is taken as an estimate for uncertainty[56]. However, this approach may be problematic as the noise introduced in the layers is arbitrarily chosen (while constrained by physical parameters) in intensity and distribution. Moreover, one could argue that this kind of estimation could be performed even in absence of the input layers as one could simply calculate the product of the different noises introduced and thus derive uncertainty. Another issue is that this approach fails to account for different sources of noise; for instance noise in estimating the texture, could come from the uncertainty about the granulometric fraction within a textural class (as used by Doetterl et al.[56]) or from the misprediction of the textural class itself.

Given previously proposed potential problems associated with the global uncertainty analysis, in this study a different approach was followed representing the uncertainty as a probability distribution through the use of a Bayesian modling technique. The idea is to use the data distribution to estimate the uncertainty in the prediction. Given that the RUSLE is based on the product, for simplicity all the layers were log-transformed. Next, each of the input layers was treated as a spatial random field. A random field is a stochastic process defined in terms of expectation and covariance, once these two parameters are estimated, different simulation of the

field can be created. Each of the simulation has the same parameters, but differs due to the stochasticity of the process. By combining a large number of simulations, one could, in principle, estimate how the uncertainty propagates to the model output (soil loss). As deriving spatially continuous simulations for each of the layers is impractical, a simulation approach based on Gibbs sampling and an additive model was used.

The model is expressed as:

$$z\,(S_0) = z(R) + z(LS) + z(K) + z(C) + e(s)$$

where the z() values are realization of each of the log-transformed model input layers and e(s) is the spatial component of the model.

A Markov Chain Monte Carlo (MCMC) algorithm, was used to derive realizations of $z(S_0)$ (soil loss) by simulating from the multivariate normal distribution with zero mean and covariance matrix Vb, where Vb is the Bayesian covariance matrix of the fitted model. MCMC was applied using the JAGS software[58] through R interface[59].

The map of uncertainty is presented in Supplementary Fig. 8 as the standard deviation of the MCMC simulated values. The map gives an outline of the geographical distribution of the prediction variance, and it can be used to compare the potential error in different areas of the world. As also observed by Teng et al.[60] in a large-scale analysis in Australia, a tendency of the uncertainties to be lower in areas with denser vegetation cover was found. By contrast, a tendency to higher uncertainty appears in scarcely vegetated area in arid and semiarid regions (e.g., Western Sectors of North and South America, Turkestan and central Asia) but also in areas subject to higher erosion rates such as agricultural area of US, Ethiopia, China, India and Mediterranean Europe.

The error of the model estimates associated with the input data assessed with the proposed MCMC approach is about 8 Pg yr^{-1} for the whole world. The value was calculated by calculating sample quantiles on the simulated data. It should be noted that the error interval is not symmetric around the mean, so the upper error limit (at 0.9 CI) is about 5.6 Pg yr^{-1}, while the lower is narrower at 2.4 Pg yr^{-1}. Nevertheless, the absolute value of the standard deviation has to be taken with caution as the underlying distribution is not normal, the standard deviation cannot be directly used to derive information such as confidence intervals.

Supplementary Note 5 | Model performance evaluation

To evaluate if our model outcomes comply with the regional findings of former studies, we compared the global soil erosion maps of 2001 and 2012 with a set of representative and highly advanced regional soil erosion assessments. We selected the cropland areas of the United States and Europe for a further model performance assessment because regional crop statistics, accurate soil services information and rigorous estimates of rainfall-runoff erosivity exist for these areas and were integrated into regional studies that served as adequate comparison groups. The first regional comparison was carried out for the cropland of the United States. According to the National Resources Inventory (NRI) of the US Department of Agriculture[61] the area has undergone a soil erosion decrease from 1.59 Pg yr^{-1} in 1982 to 0.960 Pg yr^{-1} in 2012. Our model predicts a soil erosion of 1.44 Pg yr^{-1} for the United States in 2001. Knowing that the US cropland decreased by 5.9% between 1982 and 2001, we applied our model on the cropland surface in 1982 and predicted a soil erosion of about 1.52 Pg yr^{-1} (considering conservation agriculture covering about 2.5% of the USA cropland in 1982). The deviation between the statistical data of the USDA and our model is only -4.4% which supports our approach. The USDA also reports a decrease of 44% in the soil erosion between 1982 and 2012 due to the implementation of soil conservation practices as well as an overall reduction of cropland area. For the comparative period, our model suggests a decrease of 27.2% driven by land use change. On top of this, we estimated an additional reduction of 17.2% in the soil conservation scenario as a result of soil conservation practices. This adds up to an overall decrease of 44.4% of our predicted values between 1982 and 2012 and totals an erosion of 0.91 Pg yr^{-1}. In fact, this constitutes a difference of only 4.5% compared to the USDA data. We performed further validity assessments comparing the results of our global soil erosion estimates with the modelled estimates of the Joint Research Centre (JRC) of the European Commission[62]. This European validity assessment also showed a broad consistency of the soil erosion estimates for the European cropland in 2012 (global = 0.304 Pg yr^{-1}; JRC = 0.30 $Pgyr^{-1}$) with a difference of only 1.1%. The soil erosion estimates of our global model are slightly lower than the ones of the JRC for Europe (global = 0.28 Pg yr^{-1}; JRC = 0.68 Pg yr^{-1}) which can mainly be attributed to the different nature of the land cover maps (CORINE land cover (EEA[63])) and the approach improvements presented in this study, i.e., the consideration of isolated shrubs and trees.

Further support for the validity of the model predictions comes from the comparison of our results with empirical data. The most extensive collection of soil erosion data was performed and

published by Montgomery[6]. Adapting the figure he created (Fig. 6), we can superimpose the results from our global analysis for different land covers, to which we have added data on native forests and data from other meta-analysis studies. Our modelling results tend to form a lower bound for measured values on cropland and the highlands, mid-range for the bare soil areas and forests, and an upper bound for cratons. The lower bounds for the cropland and highlands could be due to study bias, with erosion measurement studies tending to be reported for areas with erosion problems or even on erosion hot spots. In alpine areas it is hard to predict soil erosion as it is easily conflated with geological erosion. Moreover, snow is a further complicating factor as this covers the soils for a proportion of the year. Estimates for forest and bare soils appear reasonable, while in cratons our estimate is at the upper end. Erosion for cratons tends to be based on estimates of river sediment flux whereby it is assumed that only ca. 10% of soil eroded is actually transported to the ocean from surface water[39]. Assuming a median erosion rate of 0.001 (mm yr^{-1}) for cratons calculated from sediment fluxes and our estimated rate of soil erosion of 0.017 (mm yr^{-1}), the ratio is 6%, similar to the 10% estimated by Walling[39]. The semi-natural vegetation (mainly grassland and shrublands) sits between the forests and croplands as we might expect.

Further insights in support of the validity of the global model estimates were gained by comparing its spatial patterns of soil erosion with the ones reported by previous global studies on soil erosion and land degradation. The first comparison was conducted with the expert-based Global Assessment of Human-induced Soil Degradation (GLASOD)[64] (Supplementary Fig. 4a-b). Results show a good to fair spatial agreement for the land area located between the 50°S and 50°N. At higher latitudes, the global model predicts low erosion rates (<1 Mg ha^{-1} yr^{-1}) driven by low rainfall-runoff erosivity[52], while GLASOD reports sizable regions with medium (W2) to high (W3) water erosion damages. GLASOD is a 1:10 million-scale map considering the type, extent, degree, rate and causes of degradation within physiographic units. GLASOD was obtained from the combination of data provided by more than 300 scientists from several countries. Although qualitative in nature, and potentially affected by the different conceptualization of the degrees of degradation of the contribution scientist[65], it is still based on extensive field observations. The good agreement between the patterns of the global model and the independent GLASOD map provides supportive insights on the predictive capacity of the model. Nevertheless, GLASOD refers to observations made during the 1980s. Important global land use changes, however, occurred in the following decades[66,67] which GLASOD is not able to reflect. A second comparison

was conducted using the status of land degradation reported by the Global Assessment of Land Degradation and Improvement (GLADA)[68] (Supplementary Fig. 4c-d). The GLADA assessment, is based on remote sensing time series analyses of the normalised difference vegetation index (NDVI) for the period 1981-2003. It uses net primary productivity (NPP) as a proxy for describing land degradation. Although it does not deal directly with soil erosion, a correlation between land degradation and soil erosion exists[69]. In addition, the time-series used to assess the land status chronologically covers the period between GLASOD observations and the model of the study at hand. The soil erosion predicted for the global croplands in the scenario for the year 2001 and the spatially equivalent GLADA data were classified (both ranked into four classes using the quantile classification method) and compared. Lands already reported by GLASOD as damaged by water erosion were masked out (grey colour in Supplementary Fig. 4c-d). A remarkable agreement between the results of the two independent global assessments was observed, particularly noticeable in Central and South America, Sub-Saharan Africa, Oceania, Eastern United States, Eastern India, Eastern Europe and Japan. By contrast, discording patterns were observed in Southern Canada where GLADA reports major decreases in NPP.

In a final comparison, the measured soil erosion rates from 2500 locations across the word were superimposed to the estimates of the global model (Supplementary Fig. 15). The measured data were extracted from García-Ruiz *et al*.'s[70] meta-analysis and processed in GIS. Due to the heterogeneous nature of measurements (plot, stream sediment monitoring, reservoir and lake silting, hillslope measurements and 137Cs) covering different time periods and time resolutions and scale dependency of the soil erosion process[71,72], this comparison is presented for the sole purpose of indicating the spatial distribution of soil erosion measurements across the globe and the relative magnitude of the process. While North America and Europe appear fairly covered by soil erosion measurements and experiments the same does not hold for large parts of Africa, Asia and South America (see Supplementary Fig. 15). In these regions, the global model identified some potential hotspots and areas of concern where according to meta-analysis[70] no measurements are available, providing insights for a more strategic approach in directing new monitoring/ modelling efforts.

Supplementary Methods

Global RUSLE based model. RUSLE is an empirical model which belongs to the detachment-limited model type. Although the overland flow may theoretically transport an infinite sediment amount, the quantity of sediment available to be moved is actually limited by the soil detachment capacity defined by the erosivity of the rainfall. The soil erosion (Mg ha^{-1} yr^{-1}) resulting from sheet and rill erosion processes is given by the following multiplicative equation:

$$A = R \cdot L \cdot S \cdot K \cdot C \cdot P$$

Equation 1

where: A (Mg ha^{-1} yr^{-1}) is the annual average soil erosion, R (MJ mm h^{-1} ha^{-1} yr^{-1}) is the rainfall-runoff erosivity factor, K (Mg h MJ^{-1} mm^{-1}) is the soil erodibility factor, L (dimensionless) is the slope length factor, S (dimensionless) is the slope steepness factor, C (dimensionless) is the land cover and management factor, P (dimensionless) is the soil conservation or prevention practices factor.

Model Parameters. The RUSLE model is structured as multiplicative equation counting six environmental factors, which were computed as follow.

Land Cover and Management Factor. The C-factor (Supplementary Fig. 10) measures the combined effect of all the interrelated cover and management variables on the soil erosion process[73]. For this global assessment, we followed the path paved in previous national[74] and pan-European[62] studies. Accordingly, two different approaches were undertaken to estimate C-factor values for agricultural and non-agricultural land.

C-factor for the agricultural land. We used a twelve-year of crop harvested area to statistically describe the crop rotations (2001-2012) of each country, while a set of 170 crops was considered. The statistical data were collected at national level from the Food and Agriculture Organization's (FAO) FAOSTAT database (http://www.fao.org/faostat/en/#data). In the following stage of data processing the 170 crops were categorized into fourteen crop groups according to their soil cover effectiveness (Supplementary Data 1). We assigned C-factor values to each of the fourteen crop groups according to literature thresholds[73,75,76] (Supplementary Table 2) to statistically describe typical crop rotations in each country.

In the following stage of data processing, the C-factor values (C_{CROP}) for each of the considered 3,252 sub-national administrative unites of the 202 countries[76] (Supplementary Fig. 11) were estimated as follow:

$$C_{CROP} = \sum_{n=1}^{14} C_{CROPn} \cdot [\%]Region_{CROPn}$$

Equation 2

where C_{CROP} represents the C-factor of the n-crop and $[\%]Region_{CROPn}$ represents the share of this crop in the agricultural land of the given region. The shares (%) of the regional crops for the rescaling operation were assessed through a statistical downscaling of the national crop statistics using the harvested areas proposed by Monfreda *et al*.[77] combining national, state, and county level census statistics with remote sensing data (for 15,990 administrative units)[78]. The data, provided in grid format by the EarthStat database (http://www.earthstat.org/), reported the global harvested areas for each of the 170 FAO crops[77] for the year 2000 with a spatial resolution of about 10x10 km. Afterwards, the regional C_{CROP} values were attributed to the IGBP-12 and IGBP-14 classes of the year 2001 and 2012. Accordingly, both maps had the same regional C_{CROP} values but their spatial extend differed due to the land use change which occurred within the considered period.

C-factor for the non-agricultural land. For the eleven IGBP classes which reported natural vegetation, the C-factor values were defined at pixel-level through a semi-qualitative approach combining a constraining range of C-factor values obtained from literature (Supplementary Table 1) with a sub-pixel-level annual representation of the average surface vegetation cover. Accordingly, the impact of the vegetation cover in the C-factor estimations was quantified by a proxy vegetation layer obtained from MODIS imagery. The surface vegetation patterns were globally described by means of the MOD44B Vegetation Continuous Fields product (VCF). This is a ca. 250m spatial resolution biophysical parameter derived from the MODIS satellite. It reports annual estimates of the percentages of i) surface vegetation cover, ii) bare soil and iii) tree cover. Data were downloaded via the NASA EarthData facility and pre-processed using the MODIS Reprojection Tool. Multiple annual data were downloaded and averaged to limit the effects of rainfall inter-annual variability on NDVI and the cloudiness variability. For the reference year 2001, the average VCF values of the years 2000, 2001 and 2002 were considered, whereas for the reference year 2012 the years 2011, 2012 and 2013 were used. Subsequently, for the areas where

no land use changes occurred the average of the 2001 and 2012 images were applied in order to further reduce potential bias related to the climate variability of the NDVI. For the area which had undergone land use change, the three year average of each reference year was used.

A preliminary land cover and management C-factor for non-agricultural land (C_P) was calculated as:

$$C_P = MIN_C + \left((MAX_C - MIN_C) \cdot NVS\right)$$

Equation 3

where the MIN_C and MAX_C values were set following the literature e[73,75,57,79–83,]. NVS (Non Vegetated Surface) was normalised to a range from 0 to 1 and described the percentage of ground covered by any vegetation type.

Within a next step, the final land cover and management C-factor for non-agricultural land (C_{NA}) was computed including the tree coverage (TC) with was normalised ranging from 0 to 1:

$$C_{NA} = MIN_{CF} + \left((MAX_{CF} - MIN_{CF}) \cdot TC\right)$$

Equation 4

where the $MINC_F$ and MAX_{CF} values were set according to Wischmeier and Smith[73] as 0.0001 (100% canopy cover) and 0.009 (spare forest vegetation). From this data processing step, the forestland class was excluded to avoid redundancy.

Rainfall-runoff Erosivity. The rainfall-runoff erosivity factor (R) (Supplementary Fig. 3) was computed following the USDA RUSLE handbook 703[7] using sub-hourly (61%) and hourly pluviograph data (39%) of 3,625 meteorological stations spread across 63 countries (Supplementary Fig. 12). The stations are part of the global rainfall-runoff erosivity database GloREDa[52,84] which is the result of a data collection carried out by the European Commission Joint Research Centre in collaboration with scientists and national/regional meteorological services across 63 countries.

The R-factor value for each erosive rainstorm was computed using the USDA RIST software (Rainfall Intensity Summarisation Tool)[85]. The following criteria were used to set RIST: i) rainstorms with values greater than 12.7 mm calculated in R and ii) break down storms with less

than 1.27 mm of rain in 6 hours. The average annual rainfall-runoff erosivity value (R) (MJ mm ha^{-1} h^{-1} yr^{-1}) resulted from the equation:

$$R = \frac{1}{n} \sum_{j=1}^{n} \sum_{k=1}^{mj} (EI_{30})\ k$$

Equation 5

where EI_{30} is the rainfall-runoff erosivity of a single event k, n express the number of years observed and m_j expresses the erosive events during a given year j. Thus, the rainstorm EI_{30} is given by the storm kinetic energy (E) multiplied by its 30 min maximum intensity (I30):

$$EI_{30} = E \cdot I_{30}$$

Equation 6

where the rainfall kinetic energy (E) of 1 mm of the rain in MJ ha^{-1} mm^{-1} is computed based on Brown and Foster[86]:

$$E = 0.29 \cdot \left[1 - 0.72\ e^{(-0.05\ I_r)}\right]$$

Equation 7

where I_r as the rainfall intensity expressed in mm h^{-1}.

In the second stage of data processing, the R-factor values of those points were interpolated using the Gaussian Process Regression (GPR), a non-linear regression approach that can model non-linear relations by projecting the inputs into a higher dimensional space using basis functions, and by creating a regression model in said space. As covariates we used global climate provided in the WorldClim database (www.worldclim.org), i.e., i) average monthly precipitation, ii) average minimum and maximum monthly precipitation, iii) average monthly temperature, iv) precipitation of the wettest month, v) precipitation of the driest month, and vi) precipitation seasonality. The GPR non-linear regression approach was selected due to the large number of support covariates, their potential collinearity, and the demonstrated presence of non-linear relationships between the target variable (R-factor) and the covariates.

The Slope Length and Steepness Factor. The LS-factor (Supplementary Fig. 14) is a topographic parameter which represents the influence of the terrain on surface runoff and sediment transport

capacity. Is was calculated using the GIS-based two-dimensional terrain approach proposed by Desmet and Govers[87]:

$$L_{i,j} = \frac{(A_{i,j\text{-}in} + D^2)^{m+1} - A_{i,j\text{-}in}^{m+1}}{D^{m+2} * x_{i,j}^{m} * 22.13^{m}}$$

Equation 8

where $A_{i,j\ in}$ is the contributing area at the inlet of grid cell (i,j) measured in m^2, D is the grid cell size (metres), $X_{i,j} = \sin a_{i,j} + \cos a_{i,j}$, $a_{i,j}$ is the aspect direction of the grid cell (i,j), and m is related to the ratio β of the rill to interill erosion:

$$m = \frac{\beta}{\beta + 1}$$

Equation 9

where

$$\beta = \frac{\frac{\sin\theta}{0.0896}}{[0.56 + 3*(\sin\theta)^{0.8}]}$$

Equation 10

where θ is the slope angle in degrees.

The required topographic parameters such as slope and upslope contributing area were derived from hole-filled SRTM 3 arc-seconds (ca. 90m) Digital Elevation Model[88] for the land surface between 60° North and 56° South and ASTER GDEM v2 data products for the extreme North latitudes[89]. LS values were calculated using the Desmet and Govers[87] algorithm implemented in the System for the Automated Geoscientific Analyses (SAGA) software. Estimations of LS were to a maximum slope angle of 26.6 degrees (50%)[90]. The flow accumulation was computed using the deterministic infinity algorithm[91] and a maximum hillslope length set to 122m. The use of a 90m DEM ensured the computation of the combined LS topographical factor maintaining a scale congruent with the one used during the USLE's experimental measurements with plots having a length less than or equal to 122 m. Due to the large number of data to process, the computational operations were carried out using the SAGA interface in R (RSAGA, https://cran.r-project.org/web/packages/RSAGA/index.html). A loop was built up to process the large amount

of digital elevation data and the LS values were computed overnight. A total of 870 tiles of ca. 500 by 500 km were created. The flow accumulation was computed with the multiple flow algorithm. Finally, the LS-factor grid was i) rescaled to the values of a 25m DEM through a linear relationship ($R^2 = 0.998$) and ii) resampled to ca. 250 x 250 m spatial resolution applying the nearest resampling method in ArgGIS.

Soil Erodibility. The soil erosivity factor (K) (Supplementary Fig. 13) is an empirical parameter expressing the susceptibility of the soil to be eroded. It can be estimated by means of intrinsic topsoil properties, i.e., organic matter, texture, permeability and structure.

In this study, the topsoil properties of the ISRIC SoilGrids[92] database at 1 km spatial resolution were used. We thus calculated the global K factor values following Wischmeier and Smith's[73] textural characteristics (M: percentage of silt plus fine sand fraction content multiplied by 100 minus clay fraction), organic matter (OM, in percent), soil structure (s) and permeability classes (p):

$$K = \frac{\left(2.1 \times 10^{-4}\ M^{1.14}\ (12 - OM) + 3.25\ (s - 2) + 2.5\ (p - 3)\right)}{100} \cdot 0.137$$

Equation 11

Detachment of SOC by erosion. The soil erosion rates modelled with the RUSLE model were used to compute the soil carbon detached by erosion (C_{loss}) in 2012 (baseline scenario):

$$C_{loss} = SL \cdot (SOC/100)$$

Equation 12

where SL is the soil erosion estimated by our model (baseline scenario) and the SOC is the soil organic carbon content (%) of the surface layer (depth 0-5 cm) (250m spatial resolution) of the SoilGrids[92].

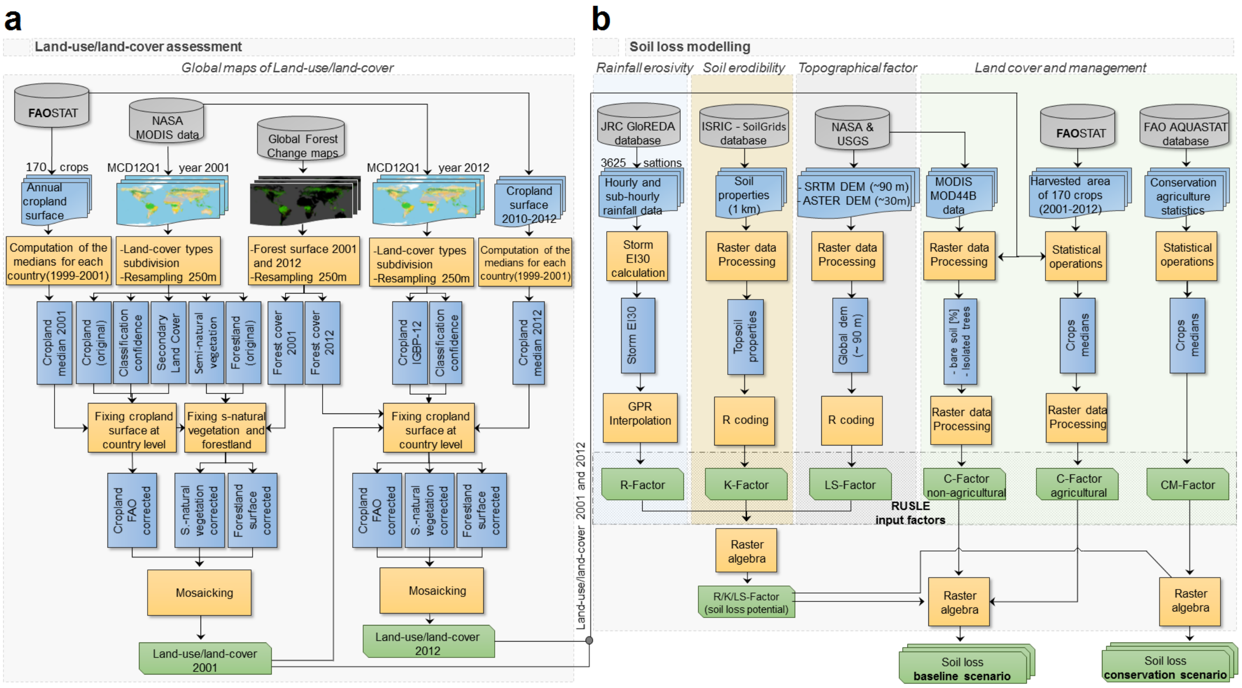

Supplementary Figure 1 Workflow of the proposed global soil erosion modelling. Schematic outline of the main processing steps carried out to **a** assess the extent, types and spatial distribution of the global croplands that are thoroughly defined combining satellite-derived land use and land cover information with agricultural inventory data, and **b** perform the high-resolution global soil erosion estimates.

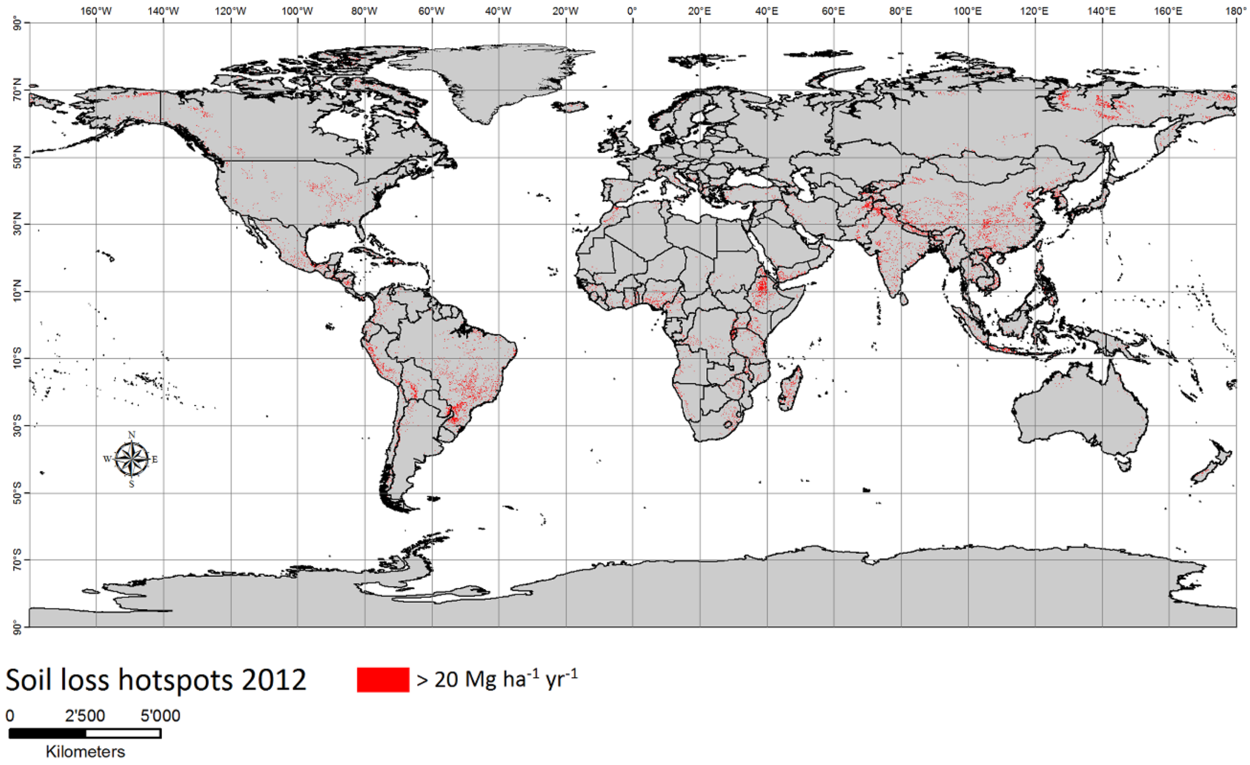

Supplementary Figure 2 Global soil erosion hot-spots. Illustration of the soil erosion hotspots (highlighted in red in the map) across the globe defined as the areas with soil erosion estimates above 20 Mg ha^{-1} yr^{-1}.

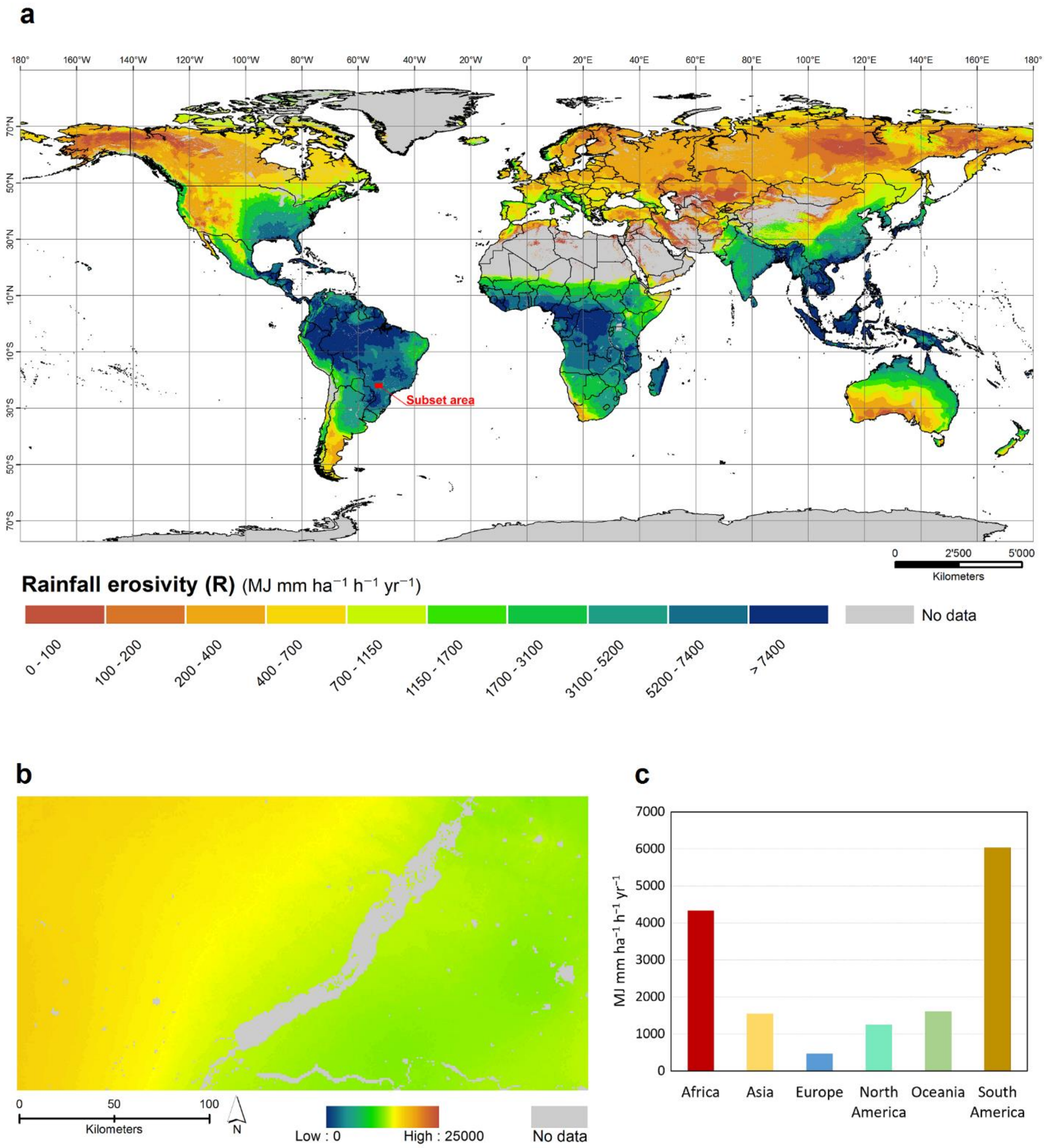

Supplementary Figure 3 Global rainfall erosivity map. **a** Representation of the global patterns of the rainfall erosivity R-factor (spatial resolution 30 arc-seconds, ca. 1 x 1km). **b** Subset of the global rainfall erosivity map for an area of about 45,000 km^2 in the West Central Region of Brazil. (**c**) R-factor average value per continent.

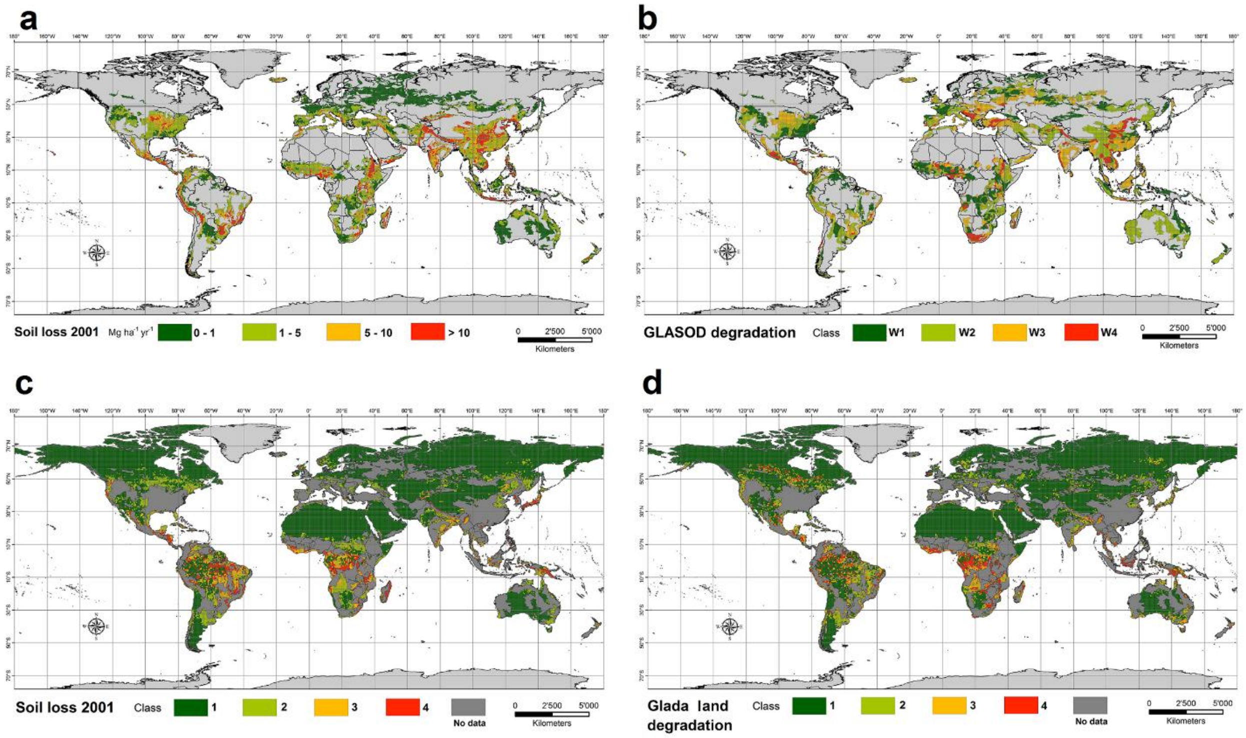

Supplementary Figure 4 Global soil erosion and land degradation assessments. **a** Representation of the average soil erosion rates for the baseline scenario 2001. Values are presented only for the areas described experiencing soil water erosion by the UNEP's project Global Assessment of Soil Degradation (GLASOD). **b** Areas affected by water according to GLASOD. The degree of damage is indicated from low (W1) to severe (W4). **c** Average soil erosion rates for the baseline scenario 2001 ranked into four classes using the quantile classification method (the colour scale indicates increasing erosion from green to red). **d** Averaged land degradation of the Global Assessment of Land Degradation and Improvement (GLADA) ranked into four classes using the quantile classification method (the colour scale shows increasing land degradation from green to red).

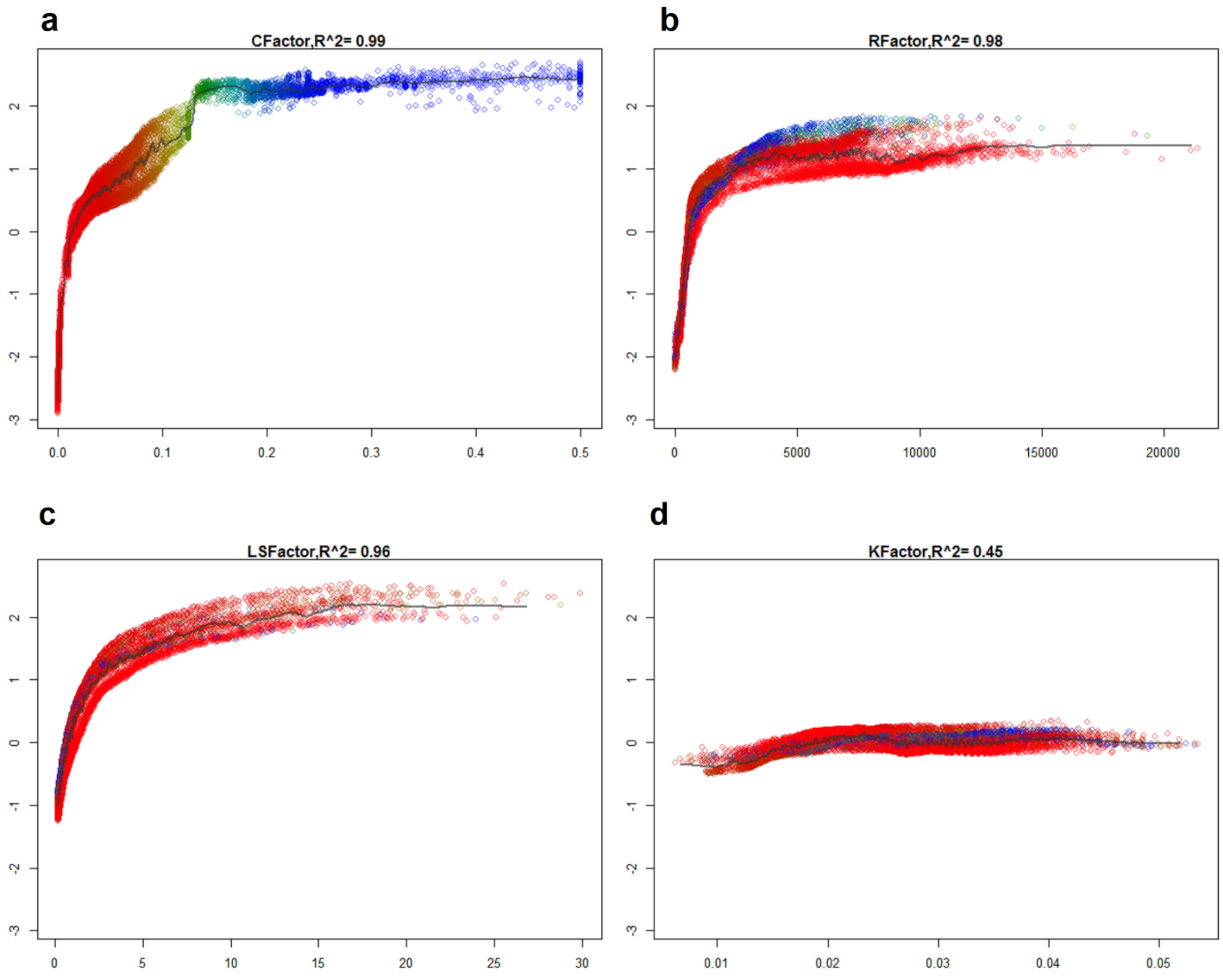

Supplementary Figure 5 Feature contribution decomposition of random forest model for the global RUSLE-based model. The model input considered is **a** the cover and management (C-factor), **b** the rainfall-runoff erosivity (R-factor) **c** the slope length and steepness factor (LS-factor) and **d** soil erodibility (K-factor). The response is expressed in logarithmic (log soil erosion) scale on the vertical axis, the independent variable is plotted on the horizontal axis. The colour scale of the four plots expresses the value of the most influential independent variable (C-factor) and allows to visualize the presence of interactions.

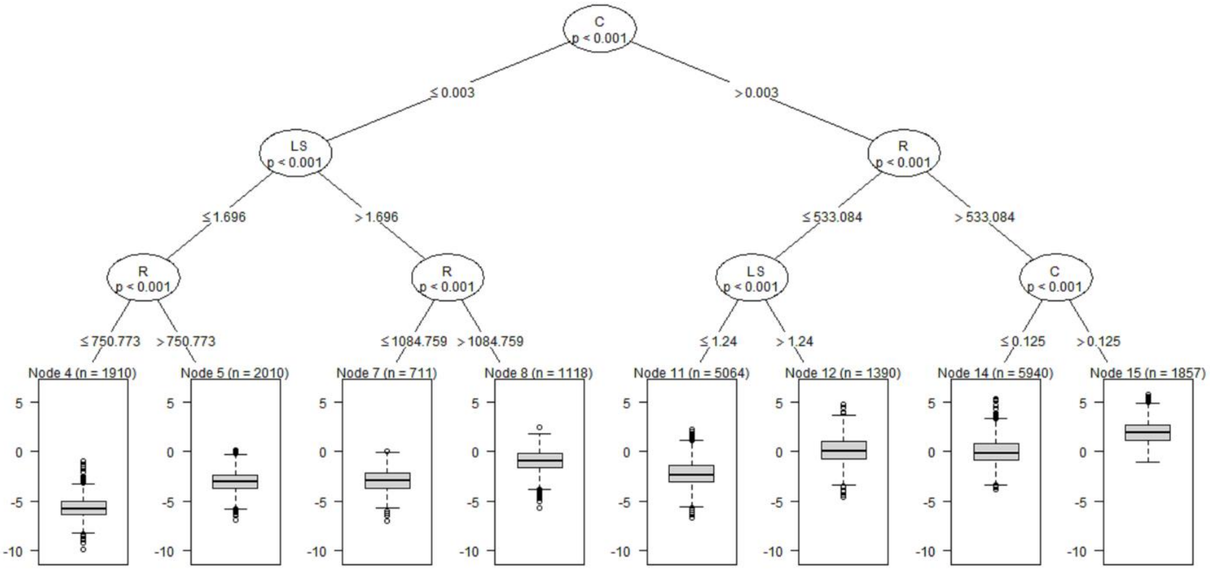

Supplementary Figure 6 RUSLE factor interactions derived from the Classification and Regression Trees (CART). The dataset is represented as a tree plot (upper part), reporting the importance of the interactions between factors with a downwards trend. The boxplot at the terminal nodes level (lower part) shows how their interaction affects the estimated soil erosion.

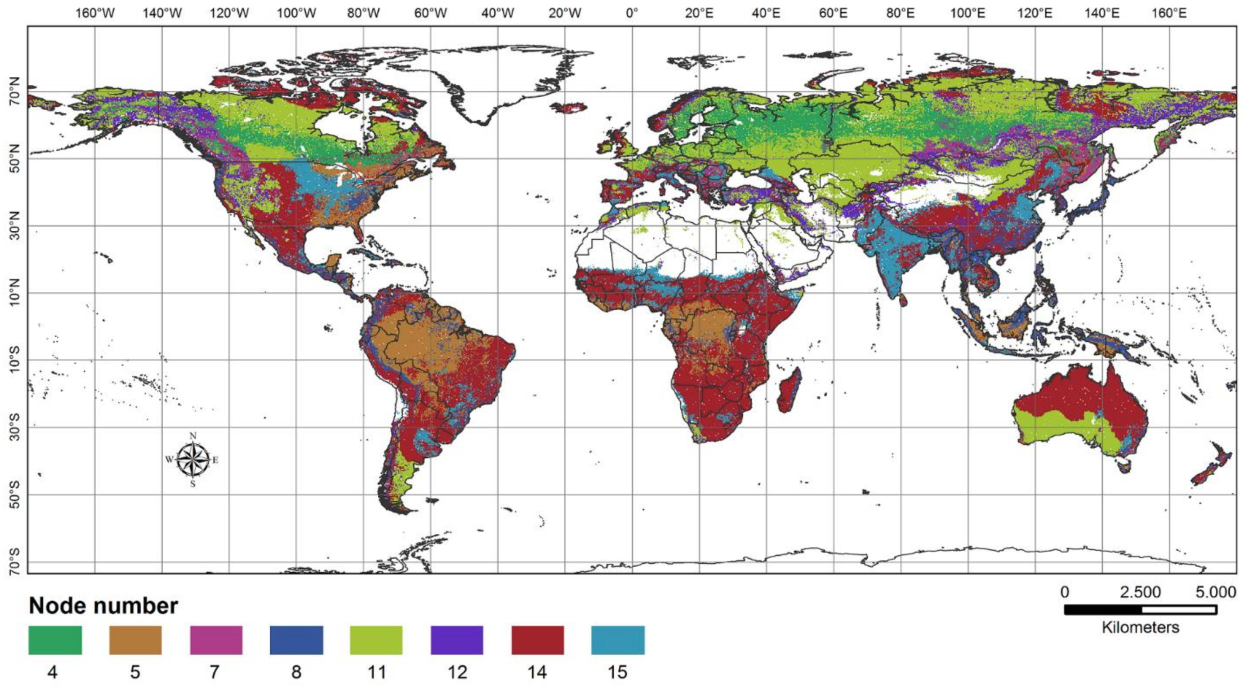

Supplementary Figure 7 Spatial representation of the node membership derived from the sensitivity analysis. It was conducted to evaluate the influence of parameter estimation on uncertainty and the effect of parameters on the global RUSLE-based model output. Each pixel in the map is attributed (colour) to the most frequent node class obtained from the Classification and Regression Trees (CART) analysis (Supplementary Note 3).

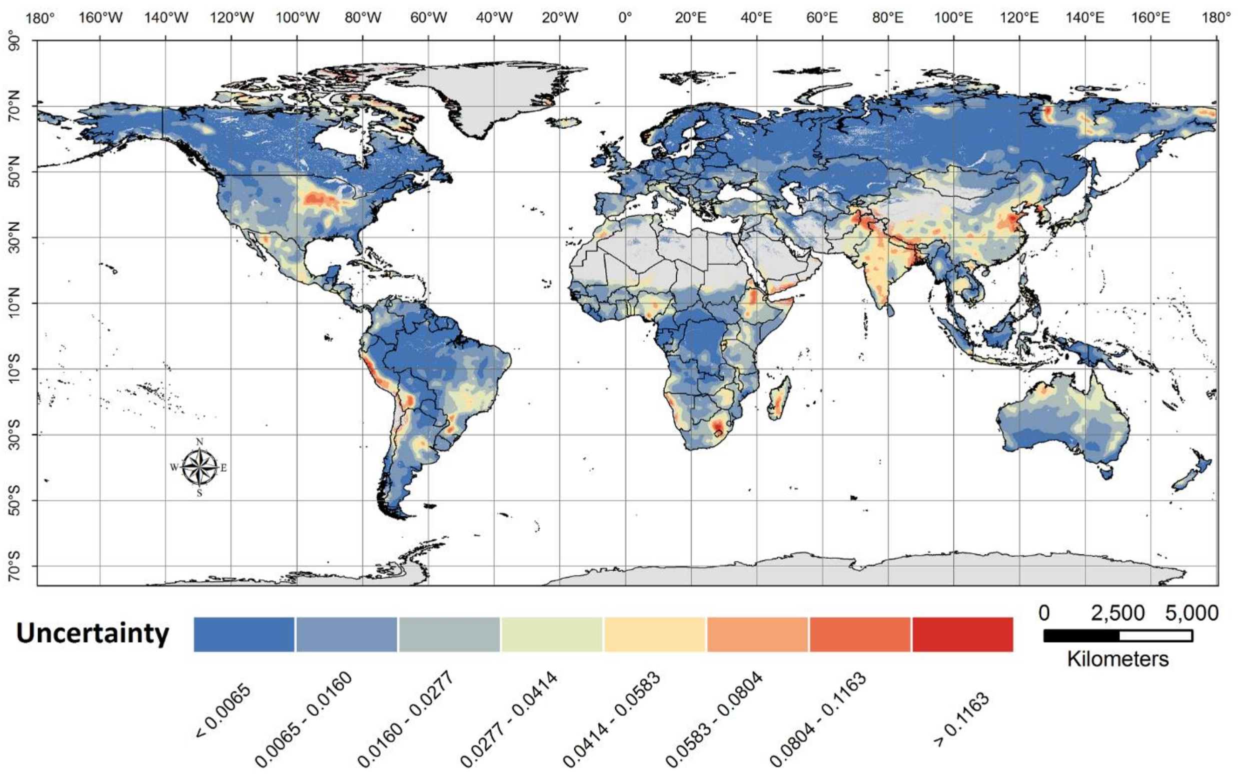

Supplementary Figure 8 Uncertainty analysis. The map of uncertainty presented as the standard deviation of the Markov Chain Monte Carlo (MCMC) simulated values.

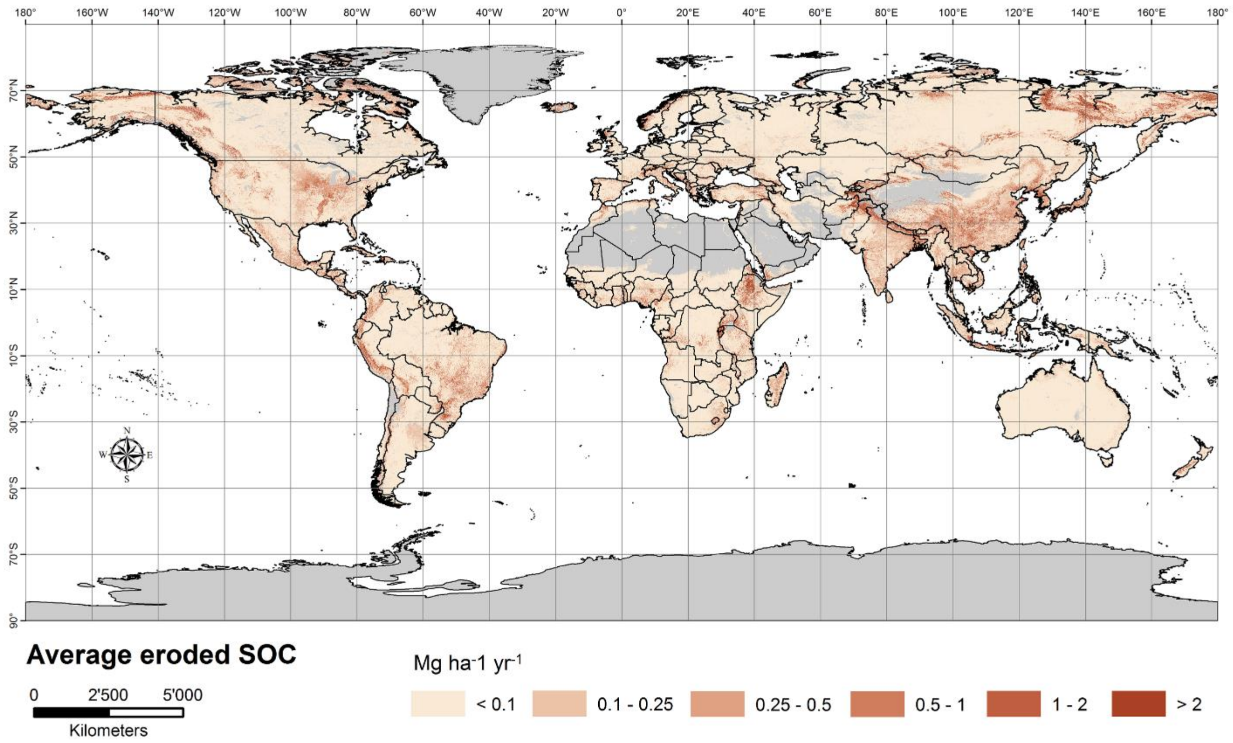

Supplementary Figure 9 Distribution of average eroded SOC for the year 2012. Values refer to the baseline scenario and are expressed in Mg ha^{-1} yr^{-1}.

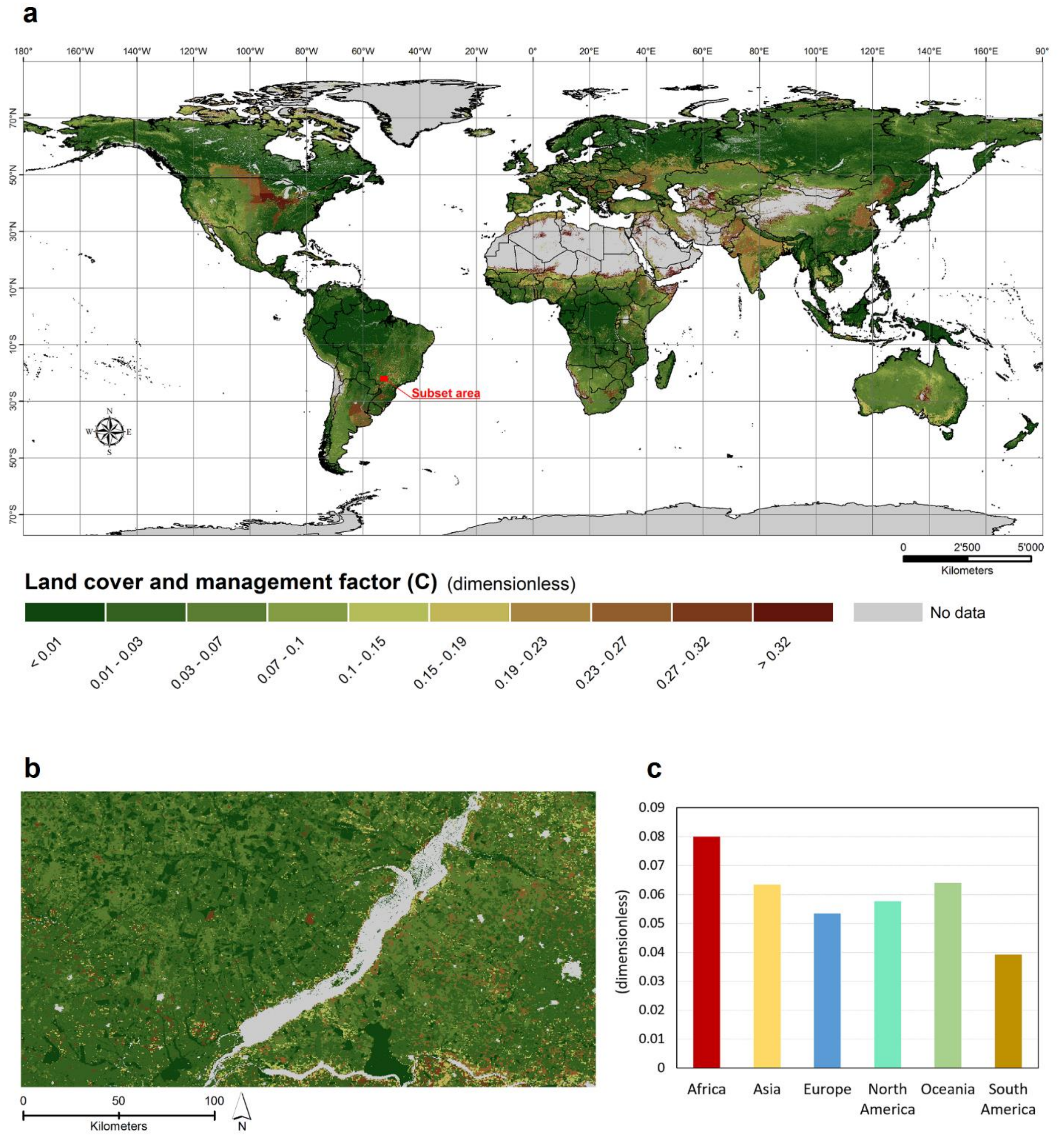

Supplementary Figure 10 Land cover and management factor (C). Figure **a** and **b** share the same legend. **a** Representation of the global patterns of the C-factor (spatial resolution ca. 250 x 250m). **b** Subset of the global C-factor map for an area of about 45,000 km^2 in the West Central Region of Brazil. **c** C-factor average value per continent.

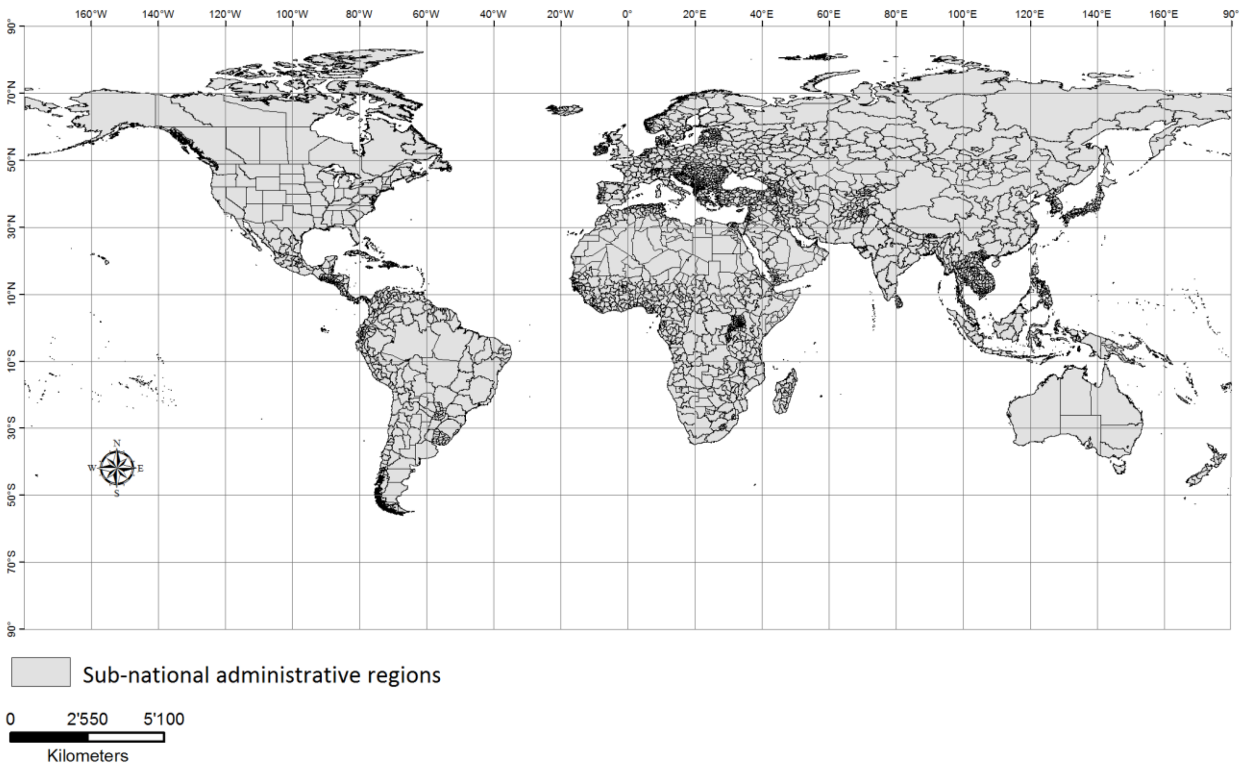

Supplementary Figure 11 The Global Administrative Unit Layers (GAUL). The data were used for the regional downscaling of the C-Factor.

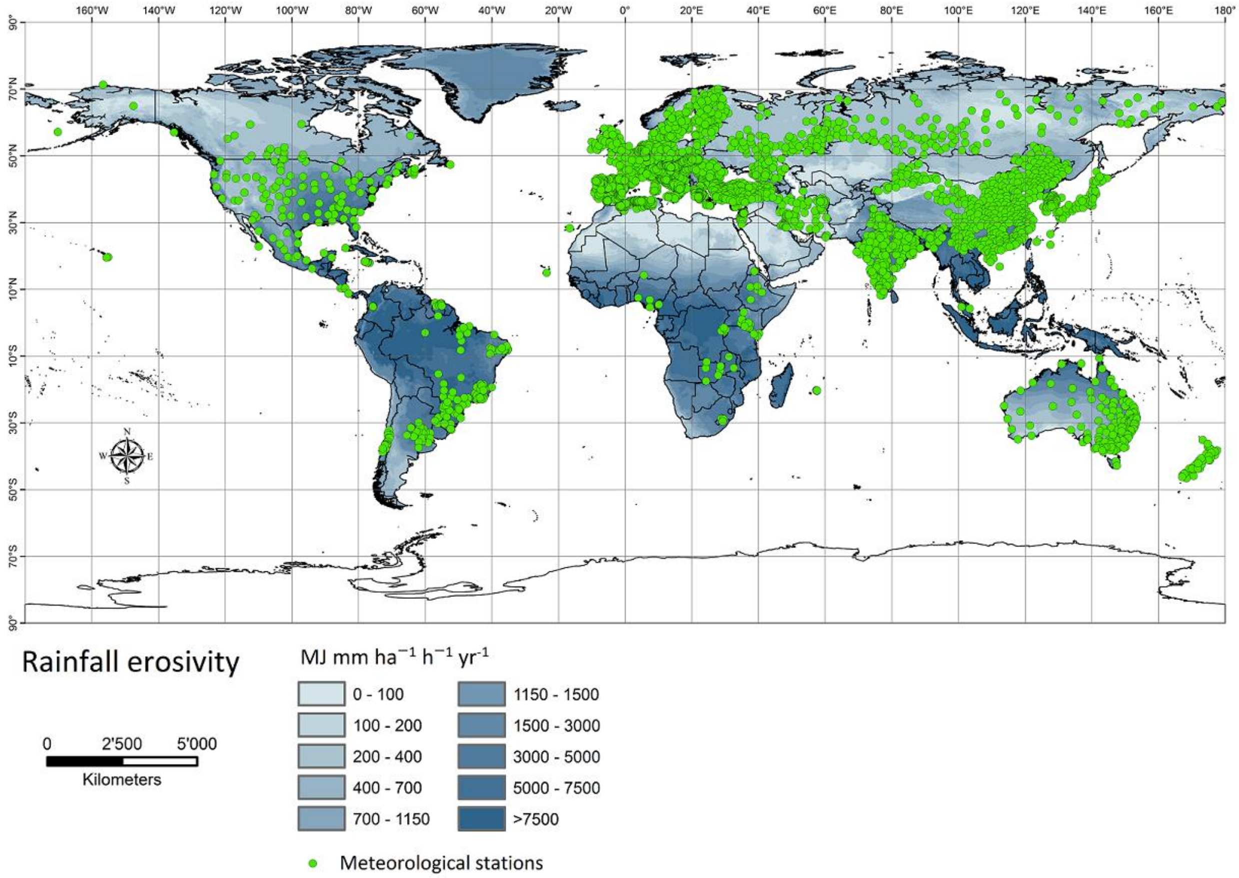

Supplementary Figure 12 Global map of the rainfall-runoff erosivity. The map was obtained through a Gaussian Process Regression (GPR) geo-statistical model interpolating hourly and sub-hourly data recorded from 3,625 stations (green dots) spread across all continents. The colour gradient from light blue to darker blue indicates the intensity of the rainfall erosivity expressed in MJ mm ha^{-1} h^{-1} yr^{-1}.

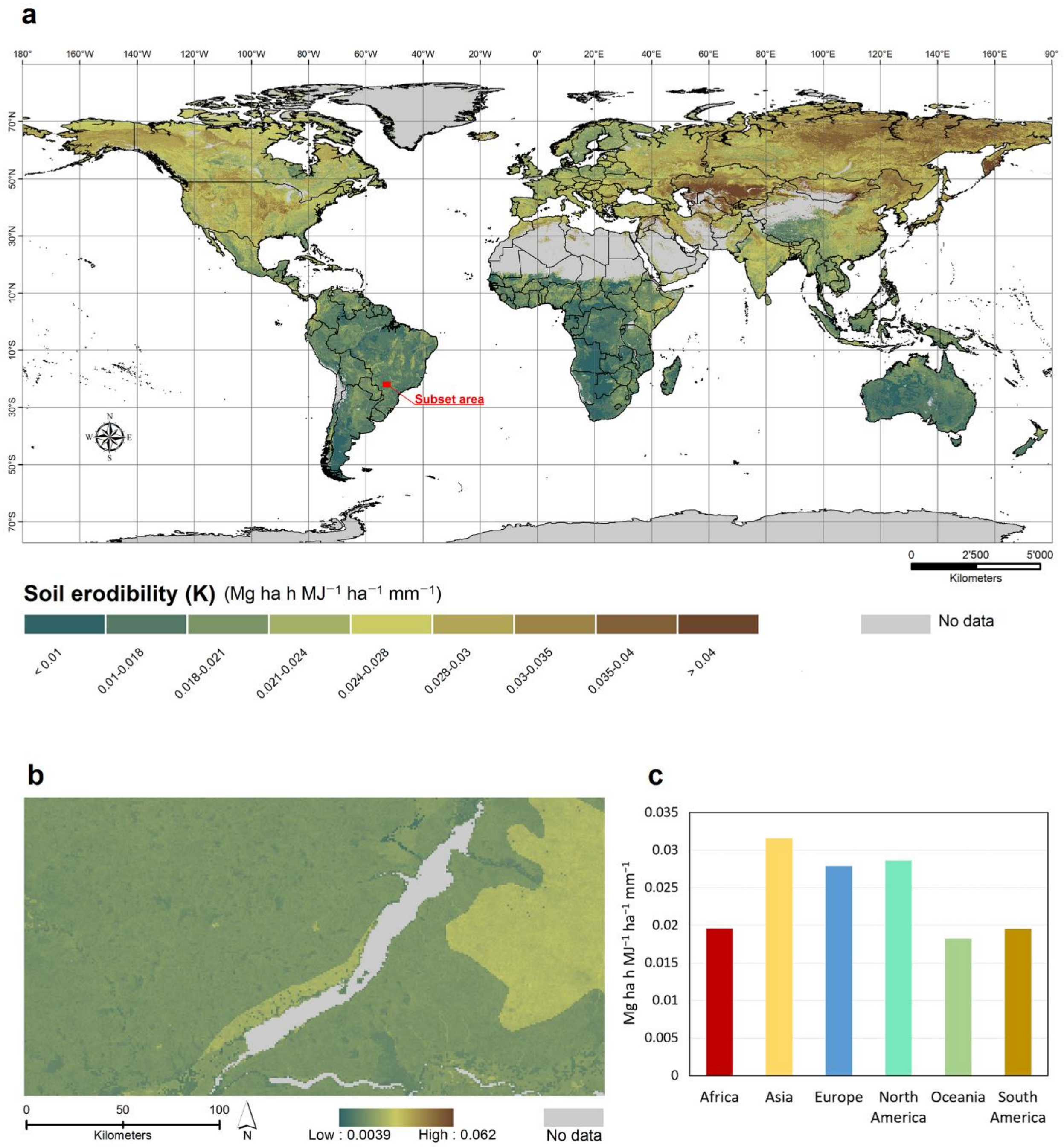

Supplementary Figure 13 The soil erodibility (K). a Representation of the global patterns of the K-factor (original spatial resolution ca. 90 x 90m resample to ca. 250 x 250m). **b** Subset of the global K-factor map for an area of about 45,000 km^2 in the West Central Region of Brazil. **c** K-factor average value per continent.

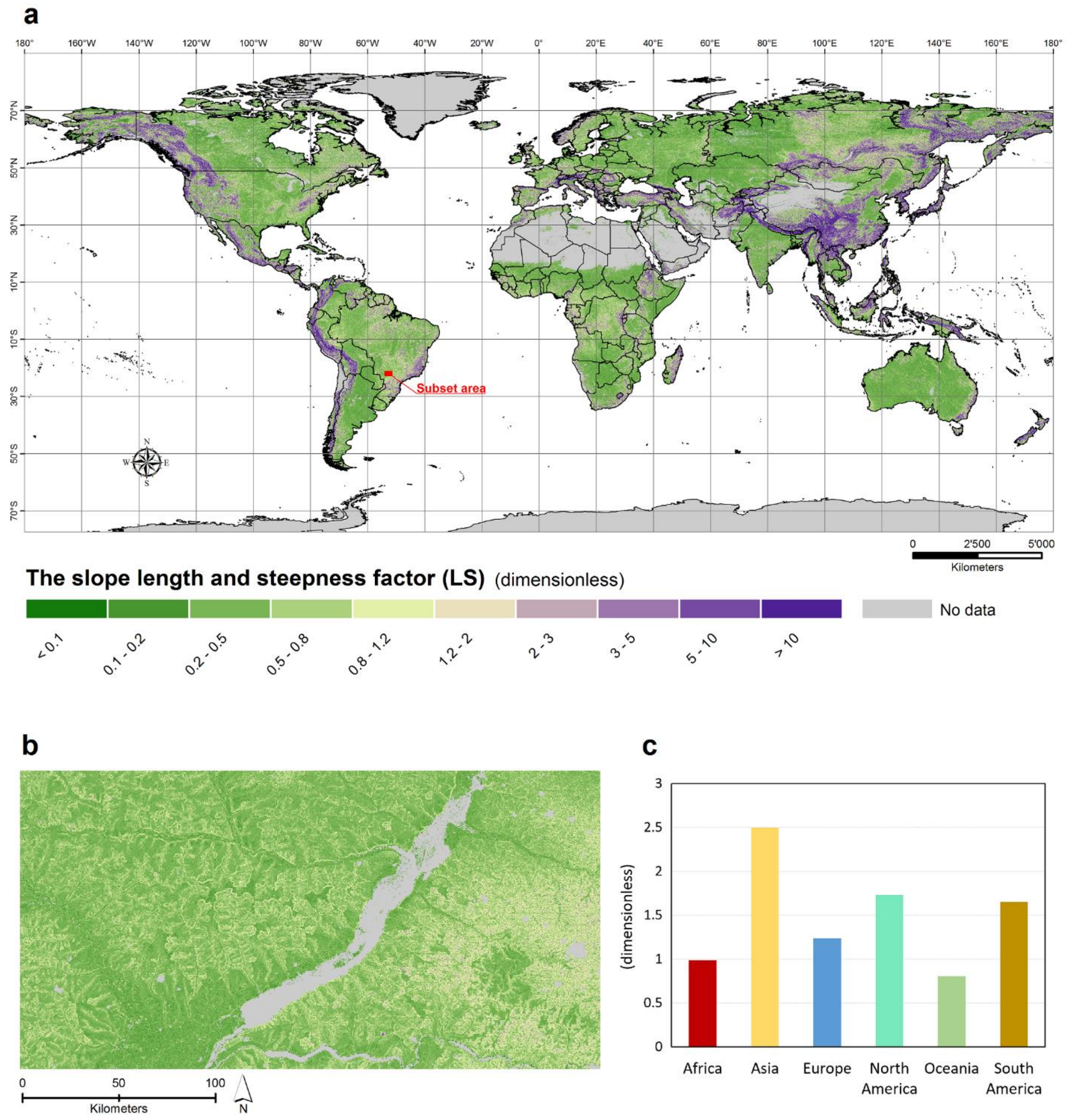

Supplementary Figure 14 The slope length and steepness factor (LS). Figure **a** and **b** share the same legend. **a** Representation of the global patterns of the LS-factor (original spatial resolution ca. 90 x 90m resample to ca. 250 x 250m). **b** Subset of the global LS-factor map for an area of about 45,000 km^2 in the West Central Region of Brazil. **c** LS-factor average value per continent.

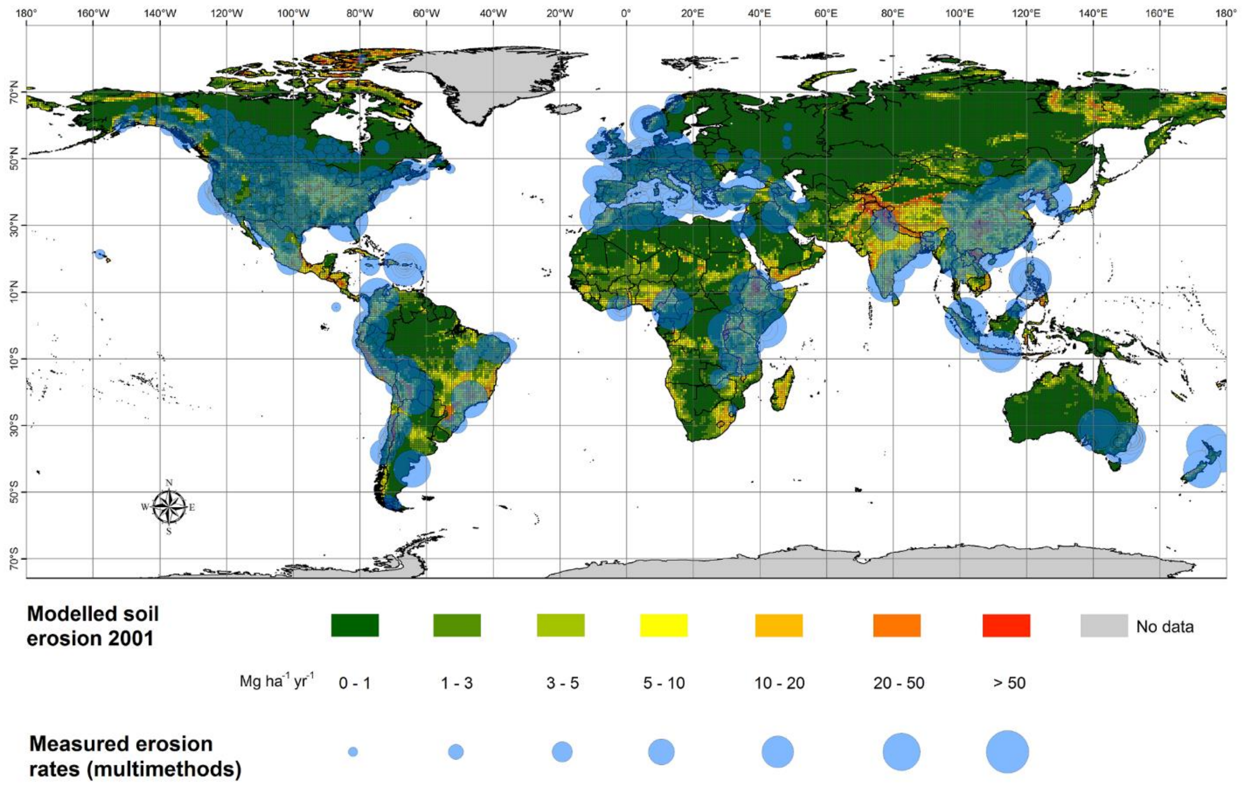

Supplementary Figure 15 Model comparison. Superimposing of measured (blued circles) versus modelled soil erosion rates (chromatic range from green to red). The measured data were extracted from the meta-analysis of García-Ruiz et al. (2015) based on different sets of measurements (plot, stream sediment monitoring, reservoir and lake silting, hillslope measurements and 137Cs). Values are all reported in Mg ha^{-1} yr^{-1}.

Supplementary Table 1 Values of C-factor for the non-agricultural land. Values are based on land use/ land cover classified according to the MODIS MCD12Q1 Land Cover Type International Geosphere Biosphere Programme (IGBP). Class 21 was created after elaborations were made in this study (See Supplementary Methods).

Class	IGBP Land-cover type	C_{NA}
0	Water	No data
1	Evergreen Needleleaf forest	0.0001-0.003
2	Evergreen Broadleaf forest	
3	Deciduous Needleleaf forest	
4	Deciduous Broadleaf forest	
5	Mixed forest	
6	Closed shrublands	0.01-0.15
7	Open shrublands	0.01-0.15
8	Woody savannas	0.01-0.15
9	Savannas	0.01-0.15
10	Grasslands	0.01-0.15
11	Permanent wetlands	No data
13	Urban and built-up	No data
15	Snow and ice	No data
16	Barren or sparsely vegetated	0.1-0.5
21	transitional woodland-shrub	0.01-0.15

Supplementary Table 2 Crop groups and their corresponding base C-factors. The values [dimensionless] derived from literature (see Supplementary Methods).

	Crop Group		C-Factor
1	Cereal Grains	Various	0.2
		Maize	0.38
		Rice	0.15
2	Legume Vegetables	Various	0.32
3	Root and Tuber Vegetables	Various	0.34
4	Fruiting Vegetables	Various	0.25
5	Cucurbit Vegetables	Various	0.25
6	Bulb Vegetable	Various	0.3
7	Leafy Vegetables	Various	0.25
		Tobacco	0.5
8	Forage, Fodder and Straw of Cereal Grains Group	Mixed-legumes	0.15
		Mixed-grasses	0.1
9	Grapes and Hops	Grapes	0.35
		Hops	0.42
10	Oilseed Group	Various	0.25
		Cotton	0.4
11	Fibre Crops	Fibre Crops	0.28
12	Berries Group	Various	0.15
		Strawberries	0.2
13	Shrubs Herbs and Spices Group	Shrubs Herbs and Spices	0.15
		Coffee	0.2
14	Trees/Fruit Tree	Various	0.15

Supplementary References

1. Webster, R. Morgan, R. P. C. *Soil Erosion and Conservation, 3rd edition* (Blackwell Publishing, Oxford, 2005).2. Toy T. J., Foster, G. R., Renard, K. G. *Soil Erosion: Processes, Prediction, Measurement, and Control*. (John Wiley & Sons, New Youk, 2002).

3. Morgan, R. P. C. & Nearing, M. A. *Handbook of Erosion Modelling* (Wiley-Blackwell, New Jersey, 2011).

4. Boardman, J. Soil erosion science: Reflections on the limitations of current approaches. *Catena* **68,** 73–86 (2006).

5. Favis-Mortlock, D. in *Environmental Modelling: Finding Simplicity in Complexity: Second Edition* (eds. Wainwright, J. & Mulligan, M.) 45–67 (John Wiley & Sons, New York, 2013).

6. Wischmeier, W., Smith, D. Predicting rainfall erosion losses: a guide to conservation planning. *U.S. Dep. Agric. Handb.* **537,** 1–69 (1978).

7. Renard, K., Foster, G., Weesies, G., McCool, D. & Yoder, D. Predicting soil erosion by water: A guide to conservation planning with the Revised Universal Soil Loss Equation (RUSLE). *Agricultural Handbook* **703,** (1997).

8. Bargarello, V. & Ferro, V. Plot-scale measurement of soil erosion at the experimental area of Sparacia (southern Italy). *Hydrol. Process.* **18,** 141–157 (2004).

9. Bagarello, V., Di Piazza, G. V., Ferro, V. & Giordano, G. Predicting unit plot soil loss in Sicily, south Italy. *Hydrol. Process.* **22,** 586–595 (2008).

10. Kinnell, P. I. A. Applying the QREI30 index within the USLE modelling environment. *Hydrol. Process.* **28,** 591–598 (2014).

11. Bagarello, V., Ferro, V. & Pampalone, V. A new version of the USLE-MM for predicting bare plot soil loss at the Sparacia (South Italy) experimental site. *Hydrol. Process.* **29,** 4210–4219 (2015).

12. Di Stefano, C., Ferro, V. & Pampalone, V. Testing the USLE-M family of models at the sparacia experimental site in South Italy. *J. Hydrol. Eng.* **22,** 5017012 (2017).

13. Cao, L., Zhang, K., Dai, H. & Liang, Y. Modeling interrill erosion on unpaved roads in the

loess plateau of China. *L. Degrad. Dev.* **26,** 825–832 (2015).

14. Gessesse, B., Bewket, W. & Bräuning, A. Model based characterization and monitoring of runoff and soil erosion in response to land use/land cover changes in the Modjo watershed, Ethiopia. *L. Degrad. Dev.* **26,** 711–724 (2015).

15. Nearing, M. A. in *Environmental Modelling: Finding Simplicity in Complexity: Second Edition* (eds. Wainwright, J. & Mulligan, M.) 365–378 (John Wiley & Sons, New York, 2013)..

16. Hann, M. J. & Morgan, R. P. C. Evaluating erosion control measures for biorestoration between the time of soil reinstatement and vegetation establishment. *Earth Surf. Process. Landforms* **31,** 589–597 (2006).

17. Wischmeier, W. H. Use and misuse of the universal soil loss equation. *J. soil water Conserv.* **3,** 5–9 (1976).

18. Smith, H. J. Application of empirical soil loss models in southern Africa: A review. *S. Afr. J. Plant & Soil*, 16, 158–163 (1999).

19. Wu, Q., Flanagan, D. C., Huang, C. & Wu, F. Estimation of USLE K values with a process-based approach. *Trans. ASABE* **60,** 159–172 (2017).

20. Laflen, J. M. & Moldenhauer, W. C. *Pioneering Soil Erosion Prediction – The USLE Story*. (World Association of Soil and Water Conservation, Beijing, 2003).

21. Risse, L. M., Nearing, M. A., Laflen, J. M. & Nicks, A. D. Error assessment in the universal soil loss equation. *Soil Sci. Soc. Am. J.* **57,** 825 (1993).

22. A. K. Tiwari, L. M. Risse & M. A. Nearing. Evaluation of WEPP and its comparison with USLE and RUSLE. *Trans. ASAE* **43,** 1129–1135 (2000).

23. Kinnell, P. I. A. Event soil loss, runoff and the Universal Soil Loss Equation family of models: A review. *J. Hydrol.* **385,** 384–397 (2010).

24. Auerswald, K., Fiener, P., Martin, W. & Elhaus, D. Use and misuse of the K factor equation in soil erosion modeling: An alternative equation for determining USLE nomograph soil erodibility values. *Catena* **118,** 220–225 (2014).

25. Arnold, J. G., Srinivasan, R., Muttiah, R. S. & Williams, J. R. Large area hydrologic

modeling and assesment Part I: Model development. *J. Am. Water Resour. Assoc.* **34,** 73–89 (1998).

26. Cronshey, R. G. & Theurer, F. D. in *Proceedings First Federal Interagency Hydrologic Modeling Conference* (Las Vegas, NV, 1998).

27. Van Rompaey, A. J. J., Verstraeten, G., Van Oost, K., Govers, G. & Poesen, J. Modelling mean annual sediment yield using a distributed approach. *Earth Surf. Process. Landforms* **26,** 1221–1236 (2001).

28. Williams, J. R., Renard, K. G. & Dyke, P. T. EPIC: a new method for assessing erosion's effect on soil productivity. *J. Soil Water Conserv.* **38,** 381–383 (1983).

29. Laflen, J. M., Elliot, W. J., Flanagan, D. C., Meyer, C. R. & Nearing, M. a. WEPP-predicting water erosion using a process-based model. *J. Soil Water Conserv.* **52,** 96 (1997).

30. De Roo, A. P. J., Offermans, R. J. E. & Cremers, N. H. D. T. LISEM: a single-event, physically based hydrological and soil erosion model for drainage basins. II: sensitivity analysis, validation and application. *Hydrol. Process.* **10,** 1119–1126 (1996).

31. Morgan, R. P. C. et al. The European soil erosion model (EUROSEM): a process-based approach for predicting soil loss from fields and small catchments. , 527-544. *Earth Surf. Process. Landforms* **23,** 527–544 (1998).

32. Kirkby, M. J. et al. The PESERA coarse scale erosion model for Europe. I. - Model rationale and implementation. *Eur. J. Soil Sci.* **59,** 1293–1306 (2008).

33. Stolpe, N. B. A comparison of the RUSLE, EPIC and WEPP erosion models as calibrated to climate and soil of south-central Chile. *Acta Agric. Scand. Sect. B - Soil Plant Sci.* **55,** 2–8 (2005).

34. Larson, W., Lindstrom, M. & Schumacher, T. The role of severe storms in soil erosion: a problem needing consideration. *J. Soil Water Conserv.* **52,** 90–95 (1997).

35. Bagarello, V. et al. Estimating the USLE soil erodibility factor in Sicily, South Italy. *Appl. Eng. Agric.* **28,** 199–206 (2012).

36. Ferro, V. Deducing the USLE mathematical structure by dimensional analysis and self-similarity theory. *Biosyst. Eng.* **106,** 216–220 (2010).

37. Barenblatt, G. I. *Similarity, Self-Similarity and Intermediate Asymptotics*. (Consultants Bureau, 1979).

38. Barenblatt, G. I. *Dimensional Analysis*. (Cambridge University Press, 1987).

39. Ferro, V. Further remarks on a distributed approach to sediment delivery. *Hydrol. Sci. J.* **42,** 633–647 (1997).

40. Ferro, V. & Porto, P. Sediment delivery distributed (SEDD) Model. *J. Hydrol. Eng.* **5,** 411–422 (2000).

41. Bagarello, V., Di Stefano, C., Ferro, V. & Pampalone, V. Predicting maximum annual values of event soil loss by USLE-type models. *Catena* **155,** 10–19 (2017).

42. Nearing, M. A., Yin, S.-Q., Borrelli, P. & Polyakov, V. O. Rainfall erosivity: An historical review. *Catena* **157,** (2017).

43. Saltelli, A., Trantola, S. & Chang, K. P.S. A Quantitative model-independent method for global sensitivity analysis of model output. **41,** (1999).

44. Wagner, H. M. Global Sensitivity Analysis. *Oper. Res.* **43,** 948–969 (1995).

45. Harper, E. B., Stella, J. C. & Fremier, A. K. Global sensitivity analysis for complex ecological models: a case study of riparian cottonwood population dynamics. *Ecol. Appl.* **21,** 1225–1240 (2011).

46. Benkobi, L., Trlica, M. J. & Smith, J. L. Evaluation of a refined surface cover subfactor for use in RUSLE. *J. Range Manag.* **47,** 74–78 (1994).

47. Ferreira, V. A., Weesies, G. A., Yoder, D. C., Foster, G. R. & Renard, K. G. The site and condition specific nature of sensitivity analysis. *J. Soil Water Conserv.* **50,** 493–497 (1995).

48. Estrada-Carmona, N., Harper, E. B., DeClerck, F. & Fremier, A. K. Quantifying model uncertainty to improve watershed-level ecosystem service quantification: a global sensitivity analysis of the RUSLE. *Int. J. Biodivers. Sci. Ecosyst. Serv. Manag.* **13,** 40–50 (2017).

49. Zhang, R. et al. Assessment of soil erosion sensitivity and analysis of sensitivity factors in the Tongbai-Dabie mountainous area of China. *Catena* **101,** 92–98 (2013).

50. Biesemans, J., Van Meirvenne, M. & Gabriels, D. Extending the RUSLE with the Monte Carlo error propagation technique to predict long term average off-site sediment accumulation. *J. Soil Water Conserv.* **55,** 35–42 (2000).

51. Falk, M. G., Denham, R. J. & Mengersen, K. L. Estimating uncertainty in the revised universal soil loss equation via bayesian melding. *J. Agric. Biol. Environ. Stat.* **15,** 20–37 (2010).

52. Panagos, P. et al. Global rainfall erosivity assessment based on high-temporal resolution rainfall records. *Sci. Rep.* **7,** 4175 (2017).

53. Wang, G Gertner, G Singh, V Shinkareva, S Parysow, P Anderson, A. Spatial and temporal prediction and uncertainty of soil loss using the revised universal soil loss equation: a case study of the rainfall–runoff erosivity R factor. *Ecol. Modell.* **153,** 143–155 (2002).

54. Parysow, P., Wang, G., Gertner, G. & Anderson, A. B. Assessing uncertainty of erodibility factor in national cooperative soil surveys: A case study at fort hood, Texas. *J. Soil Water Conserv.* **56,** 207–211 (2001).

55. Gertner, G., Wang, G., Fang, S. & Anderson, A. B. Effect and uncertainty of digital elevation model spatial resolutions on predicting the topographical factor for soil loss estimation. *J. Soil Water Conserv.* **57,** 164–174 (2002).

56. Doetterl, S., Van Oost, K. & Six, J. Towards constraining the magnitude of global agricultural sediment and soil organic carbon fluxes. *Earth Surf. Process. Landforms* **37,** 642–655 (2012).

57. Rompaey, A. J. J. Van & Govers, G. Data quality and model complexity for regional scale soil erosion prediction. *Int. J. Geogr. Inf. Sci.* **16,** 663–680 (2002).

58. Plummer, M. JAGS: A program for analysis of Bayesian graphical models using Gibbs sampling. in *Proceedings of the 3rd international workshop on distributed statistical computing* (DSC, Vienna, 2003).

59. R Development Core Team. R: A *Language and Environment for Statistical Computing* (R Foundation for Statistical Computing, Vienna, 2016).60. Teng, H. et al. Assimilating satellite imagery and visible-near infrared spectroscopy to model and map soil loss by water erosion in Australia. *Environ. Model. Softw.* **77,** 156–167 (2016).

61. U.S. Department of Agriculture. *2012 National Resources Inventory*. Available at: https://www.nrcs.usda.gov/Internet/NRCS_RCA/reports/nri_wet_md.html (2016).

62. Panagos, P. et al. The new assessment of soil loss by water erosion in Europe. *Environ. Sci. Policy* **54,** (2015).

63. Heymann, Y., Steenmans, C., Croissille, G. & Bossard, M. *CORINE Land Cover. Technical Guide.* (Official Publications of the European Communities, Brussel, 1994).

64. Oldeman, L. in *Soil Resilience and Sustainable Land Use* 19–36 (ISRIC, Wageningen,1994).

65. Sonneveld, B. G. J. S. & Dent, D. L. How good is GLASOD? *J. Environ. Manage.* **90,** 274–283 (2009).

66. Foley, J. A. et al. Solutions for a cultivated planet. *Nature* **478,** 337–342 (2011).

67. FAO. Crops production. http://faostat3.fao.org/download/Q/QC/E. (2016).

68. Bai, Z. G., Dent, D. L., Olsson, L. & Schaepman, M. E. Proxy global assessment of land degradation. *Soil Use and Management* **24,** 223–234 (2008).

69. FAO & ITPS. *The Status of the World's Soil Resources (Main Report)* (Food and Agriculture Organization of the United Nations, Rome, 2015).

70. García-Ruiz, J. M. et al. A meta-analysis of soil erosion rates across the world. *Geomorphology* **239,** 160–173 (2015).

71. De Vente, J. & Poesen, J. Predicting soil erosion and sediment yield at the basin scale : Scale issues and semi - quantitative models. *Earth Sci. Rev.* **71,** 95–125 (2005).

72. Borrelli, P., Märker, M., Panagos, P. & Schütt, B. Modeling soil erosion and river sediment yield for an intermountain drainage basin of the Central Apennines, Italy. *Catena* **114,** (2014).

73. Wischmeier, W. H. & Smith, D. D. Predicting rainfall erosion losses. *Agric. Handb.* **537,** 285–291 (1978).

74. Borrelli, P. et al. Effect of good agricultural and environmental conditions on erosion and soil organic carbon balance: A national case study. *Land use policy* **50,** (2016).

75. Panagos, P. et al. Estimating the soil erosion cover-management factor at the European

scale. *Land use policy* **48,** (2015).

76. FAO. *Global Administrative Unit Layers (GAUL)*. Available at: http://www.fao.org/geonetwork/srv/en/metadata.show?id=12691 (2016).

77. Monfreda, C., Ramankutty, N. & Foley, J. A. Farming the planet: 2. Geographic distribution of crop areas, yields, physiological types, and net primary production in the year 2000. *Global Biogeochem. Cycles* **22,** 1–19 (2008).

78. Ramankutty, N., Evan, A. T., Monfreda, C. & Foley, J. A. Farming the planet: 1. Geographic distribution of global agricultural lands in the year 2000. *Global Biogeochem. Cycles* **22,** (2008).

79. USDA. *Procedure for Computing Sheet and Rill Erosion in Project Areas*. (U.S. Department of Agriculture, Washington, 1977).

80. Walling, D. E. The evolution of sediment source fingerprinting investigations in fluvial systems. *Journal of Soils and Sediments* **13,** 1658–1675 (2013).

81. Yang, D., Kanae, S., Oki, T., Koike, T. & Musiake, K. Global potential soil erosion with reference to land use and climate changes. *Hydrol. Process.* **17,** 2913–2928 (2003).

82. Märker, M. et al. Assessment of land degradation susceptibility by scenario analysis: A case study in Southern Tuscany, Italy. *Geomorphology* **93,** 120–129 (2008).

83. Borselli, L., Cassi, P. & Torri, D. Prolegomena to sediment and flow connectivity in the landscape: A GIS and field numerical assessment. *Catena* **75,** 268–277 (2008).

84. Panagos, P. et al. Rainfall erosivity in Europe. *Science of the Total Environment* **511,** 801-814.

85. RIST. *Rainfall Intensity Summarization Tool (v3.88)*. Available at: https://www.ars.usda.gov/southeast-area/oxford-ms/national-sedimentation-laboratory/watershed-physical-processes-research/research/rist/ (2016).

86. Brown L.C. & Foster G. R. storm erosivity using idealized intensity distributions. *Am. Soc. Agric. Biol. Eng.* **30,** 0379–0386 (1987).

87. Desmet, P. & Govers, G. A GIs procedure for automatically calculating the USLE LS factor on topographically complex landscape units. *J. Soil Water Conserv.* **51,** 427–433 (1996).

88. Reuter, H. I., Nelson, A. & Jarvis, A. An evaluation of void-filling interpolation methods

for SRTM data. *Int. J. Geogr. Inf. Sci.* **21,** 983–1008 (2007).

89. Robinson, N., Regetz, J. & Guralnick, R. P. EarthEnv-DEM90: A nearly-global, void-free, multi-scale smoothed, 90m digital elevation model from fused ASTER and SRTM data. *ISPRS J. Photogramm. Remote Sens.* **87,** 57–67 (2014).

90. Panagos, P., Borrelli, P. & Meusburger, K. A new European slope length and steepness factor (LS-factor) for modeling soil erosion by water. *Geosci.* **5,** (2015).

91. Tarboton, D. G. A new method for the determination of flow directions and upslope areas in grid digital elevation models. *Water Resour. Res.* **33,** 309–319 (1997).

92. Hengl, T. et al. SoilGrids1km — Global soil information based on automated mapping. *PLoS One* **9,** e105992 (2014).
