# Supplementary material for: An assessment of the global impact of 21st century land use change on soil erosion
Source: Nat Commun. 2017 Dec 8;8:2013. doi: 10.1038/s41467-017-02142-7 (PMC5722879; doi:10.1038/s41467-017-02142-7)
Supplement: Supplementary file 3 — Description of Additional Supplementary Files [file 41467_2017_2142_MOESM3_ESM.docx]

**Description of Additional Supplementary Files**

File Name: Supplementary Data 1

Description: Set of the 170 crop statistics used for the computation of the C-factor for croplands. The crops are grouped in fourteen crop groups according to their soil cover effectiveness. Crop data were provided by the Food and Agriculture Organization (FAO) (FAOSTAT database).
